# Shade Nets Improve Gas Exchange and Chlorophyll Fluorescence in Young Avocado Trees Grown Under Mediterranean Conditions

**DOI:** 10.3390/plants14233550

**Published:** 2025-11-21

**Authors:** Maria Tasa, Eduardo Badal, Luis Bonet, María Amparo Martínez-Gimeno, Juan Gabriel Pérez-Pérez

**Affiliations:** 1 Centro para el Desarrollo de la Agricultura Sostenible, Instituto Valenciano de Investigaciones Agrarias (CDAS-IVIA), CV-315, km 10.7, 46113 Moncada, Valencia, Spain; tasa_marcana@gva.es; 2 Servicio de Tecnología del Riego, IVIA (STR-IVIA), CV-315, km 10.7, 46113 Moncada, Valencia, Spainmartinez_margimb@gva.es (M.A.M.-G.)

**Keywords:** heat stress, photoinhibition, photosynthesis, shading net, stomatal conductance

## Abstract

Avocado trees (*Persea americana* Mill.) grown in Mediterranean conditions are exposed to high temperatures and intense solar radiation during summer, factors that can severely compromise plant water status and key physiological processes. To minimize these stressful conditions, the use of shade nets is an agronomical technique that permits the creation of an optimal microclimate for crop development. Thus, the aim was to evaluate the effects of shade netting on the physiological response of young avocado trees commercially grown under Mediterranean climatic conditions. The main results showed similar circadian rhythms of plant water status under both crop systems (open-air and shaded) in both seasons. However, the use of shading nets altered the circadian rhythm of leaf gas exchange. In summer, stomatal conductance (*g_s_*) remained significantly more open after midday in shaded trees, allowing higher leaf transpiration (*E_leaf_*) and cooler leaf temperature (T_leaf_). A similar daily pattern was observed in chlorophyll a fluorescence parameters, including the effective quantum yield of photosystem II (ΦPSII) and the electron transport rate (ETR), with the lowest values occurring at midday. In shaded plants, ΦPSII and ETR remained higher after midday than in open-air, suggesting a lower photochemical inhibition of photosynthesis caused by heat stress and photoinhibition. Thus, the use of shade nets represents an agronomic alternative technique for cultivating avocados in Mediterranean climate conditions.

## 1. Introduction

Avocado (*Persea americana* Mill.) is a globally significant crop due to its economic value and health benefits. According to the Food and Agriculture Organization (FAO), global avocado cultivation reached approximately 858,152 hectares in 2021 [1], reflecting the exponential growth in demand for this fruit. Among the various avocado cultivars, ‘Hass’ is the dominant variety, accounting for approximately 90% of the global avocado trade. This variety’s popularity stems from its consistent quality, shelf life, and favourable organoleptic properties. In recent years, the growing European demand for avocados has prompted Mediterranean countries to expand their cultivation areas, capitalizing on lucrative export opportunities [2].

Despite its success, avocado cultivation faces unique challenges when transitioning from its native tropical and subtropical regions to Mediterranean climates. The humid, warm conditions of its native regions contrast sharply with the arid and semi-arid conditions typical of the Mediterranean basin. Mediterranean summers are characterized by high temperatures, intense solar radiation, low relative humidity, and limited water availability. When grown in Mediterranean areas, avocados face much more demanding environmental conditions, especially during the summer. As a result, these regions do not achieve the optimal production potential that the crop can express in its areas of origin, and yields are generally low and variable [3]. Furthermore, water scarcity is exacerbated in coastal regions by the potential salinity of irrigation water, posing significant challenges for crop management under these conditions. The impacts of climate change are expected to exacerbate these challenges, with increasing temperatures, prolonged drought periods, and erratic rainfall patterns as critical stressors for agriculture in Mediterranean regions [4]. For avocado, these environmental changes may further disrupt the delicate balance required to maintain optimal growth and yield under Mediterranean conditions.

Avocado is characterized by isohydric behaviour [5], which allows it to maintain relatively stable leaf water potential under variable environmental conditions but also restricts gas exchange when atmospheric demand is high. Its root system is shallow [3] and highly sensitive to hypoxia and salinity, limiting water uptake in dry or saline Mediterranean soils. Avocado is considered a shade-tolerant species with an adaptive advantage in colonising small canopy gaps in its native habitats [6]. Consequently, the photosynthetic light saturation point of mature leaves in field-grown trees occurs at a photon flux density (PPFD) of approximately 1100 µmol m^−2^ s^−1^ [7], making the species particularly prone to photoinhibition under intense solar radiation. These physiological traits collectively explain the avocado’s limited tolerance to heat and light stress, highlighting the importance of evaluating shading as a strategy to mitigate these limitations.

High temperatures and excessive solar radiation can disrupt key processes, such as stomatal regulation and the plant’s capacity to dissipate excess light energy, leading to photoinhibition and oxidative stress [8,9]. These challenges impair the plant’s overall photosynthetic performance, reducing its capacity to convert light energy into chemical energy effectively. These stress-induced physiological limitations underscore the need for employing adaptive cultivation techniques to mitigate the adverse effects of Mediterranean climatic conditions on avocado production.

To address these challenges, agronomic techniques such as shade netting have been increasingly adopted in Mediterranean agriculture. Shade nets create a modified microclimate that reduces solar radiation, lowers air temperature, and maintains higher relative humidity around the plant canopy [10]. By mitigating extreme environmental stressors, shade nets can improve crop water use efficiency and enhance physiological processes. For avocado, these nets have the potential to stabilize stomatal conductance, reduce heat stress, and minimize photoinhibition during peak summer conditions.

The use of shade nets has been extensively studied in various crops, demonstrating benefits such as improved water status, enhanced photosynthetic activity, and better yield stability under stress conditions [11,12,13]. Shade nets reduce incident solar radiation and temperature, creating a less stressful environment for plants. This leads to improved photosystem II (PSII) efficiency [14], helping to prevent photoinhibition and oxidative stress [15]. However, research on avocado’s physiological response to shade netting, particularly in Mediterranean climates, remains limited. A deeper understanding of how shading affects the dynamics of avocado water relations and photosynthesis is essential for optimizing its cultivation in these regions.

Given the increasing challenges posed by Mediterranean climatic conditions, it is essential to explore innovative agronomic strategies to enhance the resilience of avocado cultivation. To date, no field studies have evaluated the effects of shading on avocado cultivation, especially under Mediterranean conditions. Most available information comes from other fruit crops, and extrapolation to avocado remains uncertain. This study aims to investigate how shade nets can mitigate the adverse effects of heat stress and high solar radiation, thereby creating a more favourable microclimate for avocado trees. This represents one of the first field-based evaluations of shading effects on avocado physiology under Mediterranean conditions. The study aims to fill the current knowledge gap by assessing how shade nets influence gas exchange and photochemical efficiency in avocado trees exposed to high radiation and temperature stress. The primary objective of this research is to evaluate the physiological responses of young avocado trees under shade netting, with a focus on their water relations and photosynthetic performance. It was hypothesized that shade nets would moderate the canopy microclimate, reducing heat and radiation stress. Under shaded conditions, trees were expected to maintain higher stomatal conductance and photosynthetic efficiency, with reduced photochemical limitations on photosystem II relative to open-air trees. By analysing the daily dynamics of these processes, this study aims to provide insights into the potential of shade netting as a sustainable cultivation technique for improving avocado growth and productivity in Mediterranean climates. The findings of this study are intended to provide a physiological basis for developing management strategies that enhance the resilience and productivity of avocado orchards in Mediterranean environments.

## 2. Results

### 2.1. Environmental Conditions

**Figure 1:**
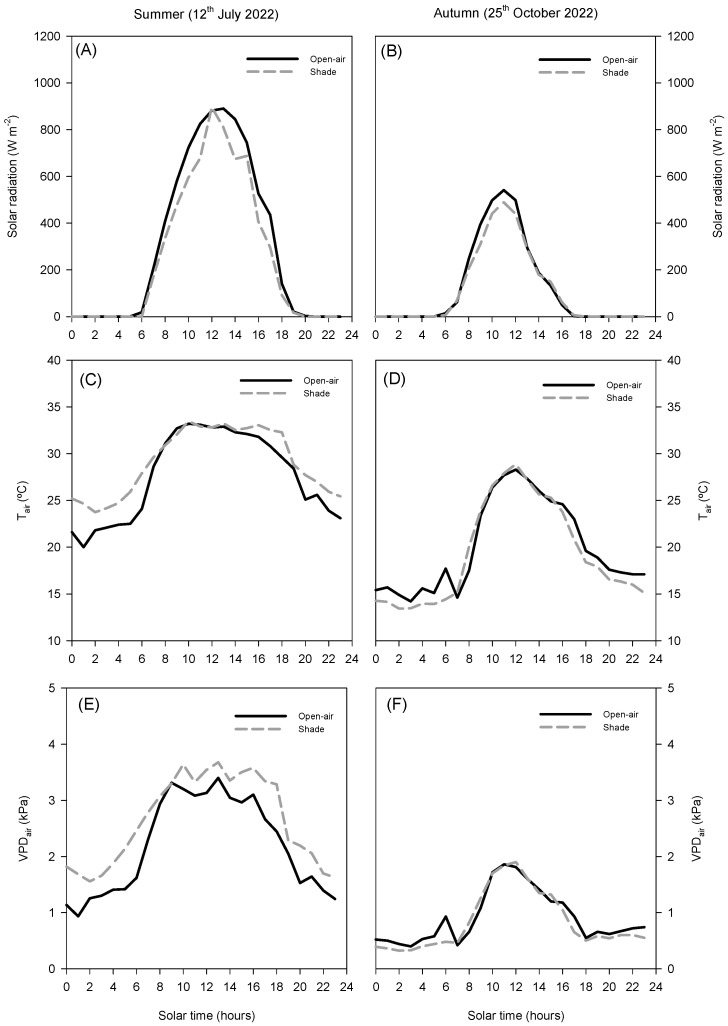
Diurnal cycle of net solar radiation (**A**,**B**), air temperature (T_air_, (**C**,**D**)), and air vapour pressure deficit (VPD_air_, (**E**,**F**)) on a representative summer day (12 July 2022) and autumn day (25 October 2022) in avocado trees grown in open-air (black line) and shade (grey line) conditions.

In summer, solar radiation reached maximum values of approximately 900 W m^−2^ at midday in open-air conditions, while shading slightly reduced peak intensity by about 10% (Figure 1A). Air temperature was similar in both systems around midday, reaching nearly 35 °C. However, during the early morning and late afternoon, shading conditions were approximately 2 °C warmer than open-air conditions (Figure 1C). The air vapour pressure deficit (VPD_air_, Figure 1D) showed slightly higher values under shade (above 3.5 kPa) compared to open-air conditions (around 3.4 kPa).

In autumn, radiation, T_air_, and VPD_air_ showed lower overall values compared to summer (Figure 1B,D,F). Solar radiation peaks did not exceed 600 W m^−2^, air temperature remained below 30 °C, and VPD remained below 2.0 kPa, with negligible differences between treatments. This seasonal reduction highlights that the impact of shading on the microclimate was more evident during summer, when plants were exposed to higher radiation and atmospheric demand, whereas in autumn, both conditions converged to similar levels.

**Figure 2:**
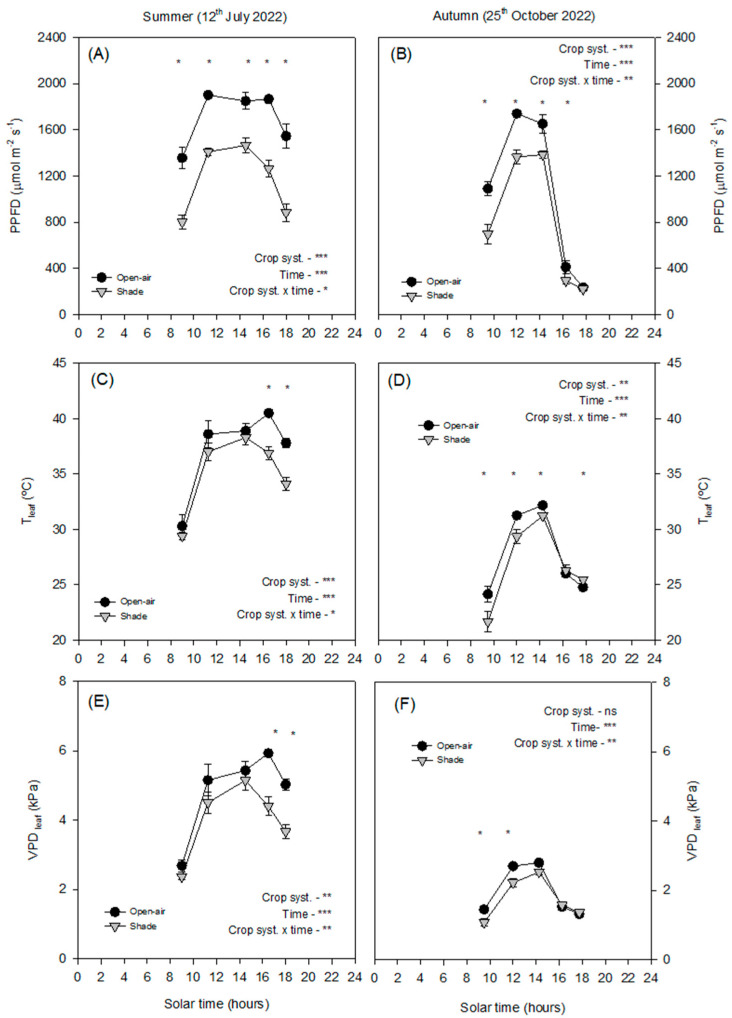
Diurnal cycle of photosynthetic photon flux density (PPFD, (**A**,**B**)), leaf temperature T_leaf_, (**C**,**D**)), and leaf vapour pressure deficit (VPD_leaf_, (**E**,**F**)) on a representative summer day (12 July 2022) and autumn day (25 October 2022) in avocado trees grown in open-air (black dots) and shade (grey dots) conditions. Vertical bars indicate ± standard error (n = 6). The *p*-values corresponding to the effects of crop system, time, and their interaction, obtained from the factorial repeated-measures ANOVA, are shown next to each variable. The symbols *, **, ***, and ‘ns’ denote *p* < 0.05, *p* < 0.01, *p* < 0.001, and non-significant differences, respectively. Asterisks placed at individual time points indicate significant differences between systems at the same hour according to the Duncan test (*p* < 0.05).

In summer, open-air trees were exposed to maximum PPFD values exceeding 2000 μmol m^−2^ s^−1^ at midday, whereas shading reduced incident radiation by ~30%, with maximum values around 1400 μmol m^−2^ s^−1^ (Figure 2A). In autumn, PPFD was lower overall, with maximum values below 1800 μmol m^−2^ s^−1^ under open-air conditions and ~1200 μmol m^−2^ s^−1^ under shade (Figure 2B).

The leaf temperature (T_leaf_) values followed a similar seasonal trend. In summer, open-air leaf temperatures reached values above 40 °C at midday, while shading consistently reduced T_leaf_ by 3–5 °C, rarely exceeding 37 °C (Figure 2C). In autumn, maximum T_leaf_ was lower in both treatments (<35 °C), although shaded leaves remained significantly cooler during midday (Figure 2D).

The VPD_leaf_ values also showed contrasting seasonal patterns. In summer, open-air plants exhibited maximum midday values of 5.5–6.0 kPa, whereas shading reduced VPD_leaf_ by ~20–25% (to ~4.0–4.5 kPa; Figure 2E). In autumn, maximum VPD_leaf_ was considerably lower (<3 kPa), with only slight differences between treatments (Figure 2F).

### 2.2. Plant Water Status and Gas Exchange

**Figure 3:**
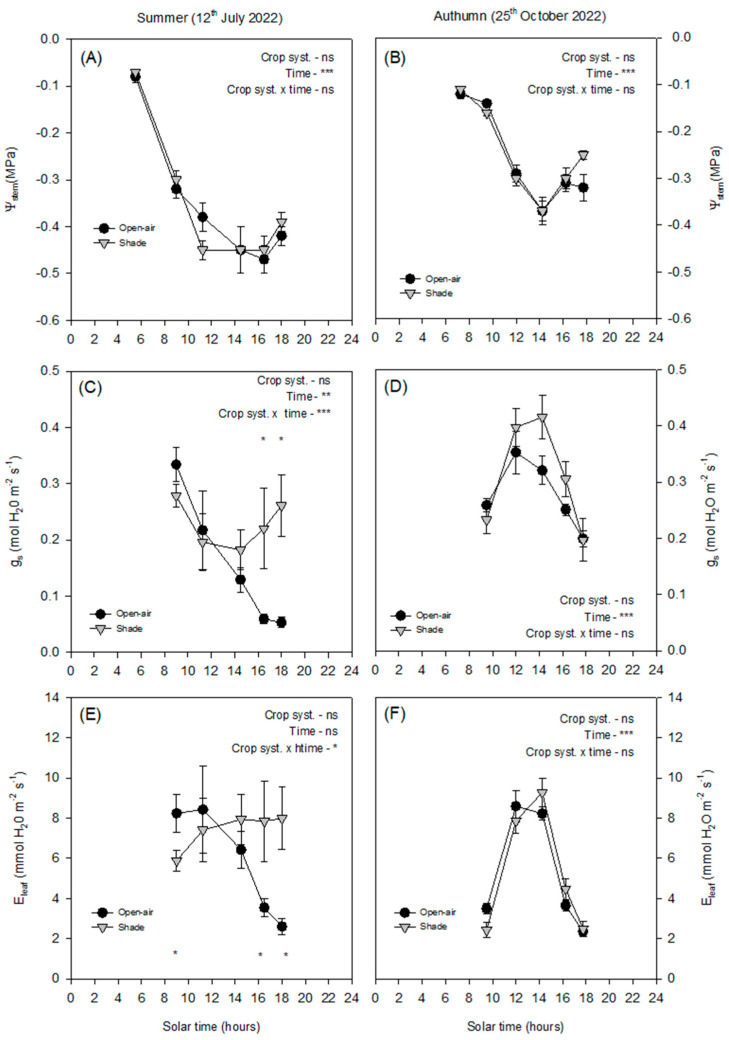
Diurnal cycle of stem water potential (Ψ_stem_, (**A**,**B**)), stomatal conductance (*g_s_*, (**C**,**D**)), and leaf transpiration (*E_leaf_*, (**E**,**F**)) on a representative summer day (12 July 2022) and autumn day (25 October 2024) in avocado trees grown in open-air (black dots) and shade (grey dots) conditions. Vertical bars indicate ± standard error (n = 6). The *p*-values corresponding to the effects of crop system, time, and their interaction, obtained from the factorial repeated-measures ANOVA, are shown next to each variable. The symbols *, **, ***, and ‘ns’ denote *p* < 0.05, *p* < 0.01, *p* < 0.001, and non-significant differences, respectively. Asterisks placed at individual time points indicate significant differences between systems at the same hour according to the Duncan test (*p* < 0.05).

In summer, Ψ_stem_ declined progressively from morning until midday, reaching minimum values close to −0.5 MPa, with no significant differences between treatments (Figure 3A), while it increased slightly in the afternoon. In summer, *g_s_* followed different diurnal dynamics in open-air and shaded plants (Figure 3C). In open-air trees, *g_s_* progressively declined from morning onwards, reaching minimum values at midday and remaining low in the afternoon. By contrast, shaded plants also showed a midday decline, but *g_s_* partially recovered later in the afternoon, resulting in significantly higher values compared with open-air conditions. Regarding *E_leaf_* (Figure 3E), open-air plants showed relatively high rates during the morning, which then dropped sharply after midday in parallel with the progressive stomatal closure. In contrast, shaded plants maintained more stable *E_leaf_* values throughout the day, with only moderate fluctuations and without the pronounced afternoon decline observed under open-air conditions.

In autumn, the Ψ_stem_ values were less negative overall (around −0.4 MPa at midday) (Figure 3B), reflecting the milder atmospheric conditions and lower demand. Both *g_s_* and *E_leaf_* showed similar trends between treatments (Figure 3D,F), with maximum values reached around midday and no consistent differences thereafter. This seasonal comparison highlights that shading exerted a stronger influence on stomatal behaviour in summer, when atmospheric demand was higher, whereas under autumn conditions, the effect was negligible.

**Figure 4:**
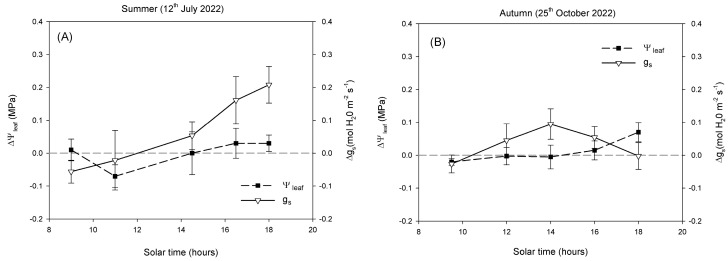
Diurnal cycle of the physiological differences between shaded and open-air trees (expressed as Δ (shade—open-air) for stem water potential (ΔΨ_stem_) and stomatal conductance (Δ*g_s_*) in a representative summer (**A**) and autumn day (**B**). The horizontal dashed line (∆ = 0) indicates no difference between systems. Values represent mean differences ± standard error (n = 6).

In summer (Figure 4A), ΔΨ_leaf_ values remained close to zero throughout the day, with small fluctuations between –0.05 and 0.05 MPa, with minimal separation between shaded and open-air trees. In contrast, Δ*g_s_* showed a progressive increase from morning to late afternoon. Values were slightly negative or close to zero in the morning, and became positive after midday, reaching maximum differences around 0.20–0.25 mol H_2_O m^−2^ s^−1^ in the evening. In autumn (Figure 4B), both ΔΨ_leaf_ and Δ*g_s_* exhibited reduced variability compared with summer. ΔΨ_leaf_ remained within a narrow range (approximately –0.02 to 0.04 MPa) during the day, with no marked hourly changes. Δ*g_s_* fluctuated modestly, with values generally between –0.05 and 0.10 mol H_2_O m^−2^ s^−1^, and a mild midday increase.

### 2.3. Leaf Chlorophyll a Fluorescence

**Figure 5:**
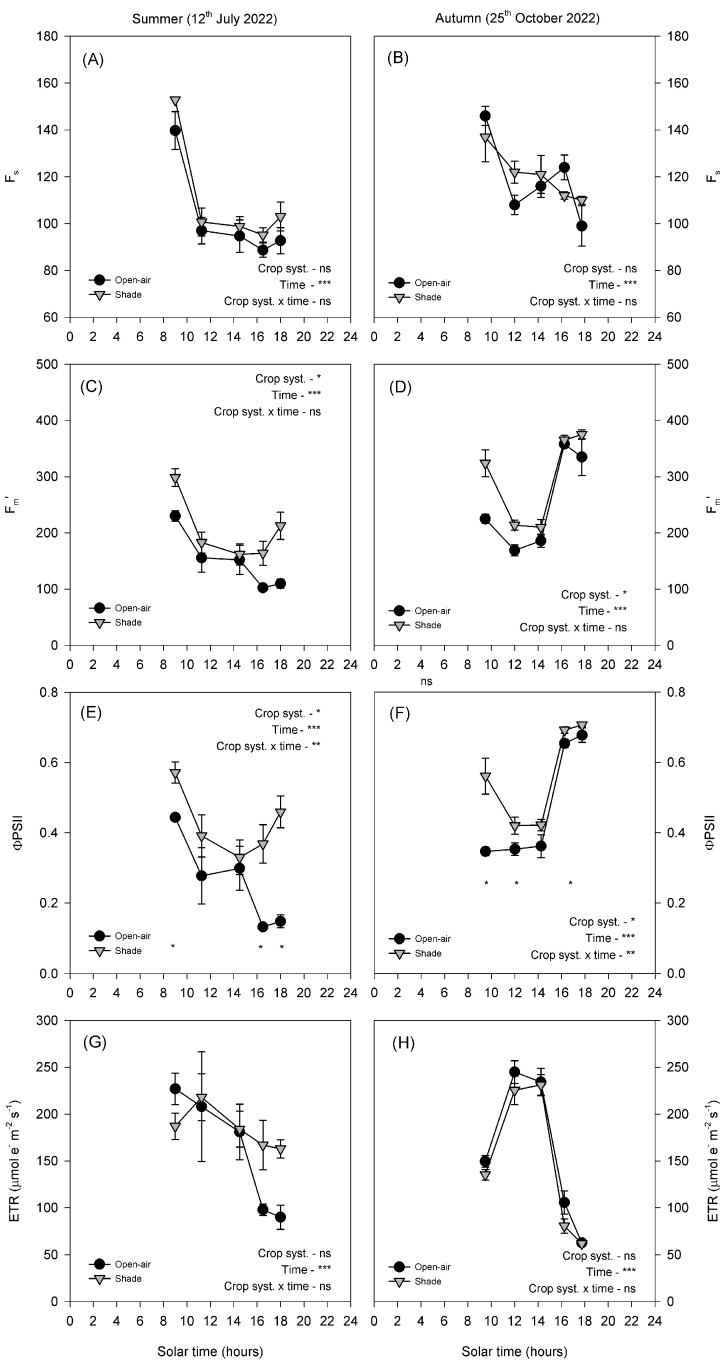
Diurnal cycle of steady-state fluorescence (*F_s_*, (**A**,**B**)), maximum fluorescence in light-adapted leaves (*F_m_*′, (**C**,**D**)), the effective quantum yield of PSII (ΦPSII, (**E**,**F**)) and the electron transport rate (ETR, (**G**,**H**)) on a representative summer day (12 July 2022) and autumn day (25 October 2022) in avocado trees grown in open-air (black dots) and shade (grey dots) conditions. Vertical bars indicate ± standard error (n = 6). The *p*-values corresponding to the effects of crop system, time, and their interaction, obtained from the factorial repeated-measures ANOVA, are shown next to each variable. The symbols *, **, ***, and ‘ns’ denote *p* < 0.05, *p* < 0.01, *p* < 0.001, and non-significant differences, respectively. Asterisks placed at individual time points indicate significant differences between systems at the same hour according to the Duncan test (*p* < 0.05).

In summer, fluorescence parameters showed clear differences between treatments (Figure 5A,C,E,G). *F_s_* and *F_m_*′ decreased markedly at midday in both conditions (Figure 5C). ΦPSII exhibited a sharp midday depression in open-air plants, which persisted throughout the afternoon. In contrast, shaded leaves partially recovered after midday, resulting in significantly higher values later in the day (Figure 5E). ETR decreased progressively throughout the day in both treatments, with no significant differences between treatments.

In autumn, both treatments exhibited higher overall fluorescence values than in summer (Figure 5B,D,F,H). *F_s_* and *F_m_*′ followed a similar diurnal course in both conditions, with shaded plants showing slightly higher values in the early morning (Figure 5D,F). ΦPSII declined strongly at midday in both treatments, although shaded leaves maintained higher values during the morning and early afternoon. ETR displayed a typical diurnal pattern, characterised by a midday peak and a subsequent decline, with no significant differences between treatments (Figure 5H).

### 2.4. Physiological Correlations

**Figure 6:**
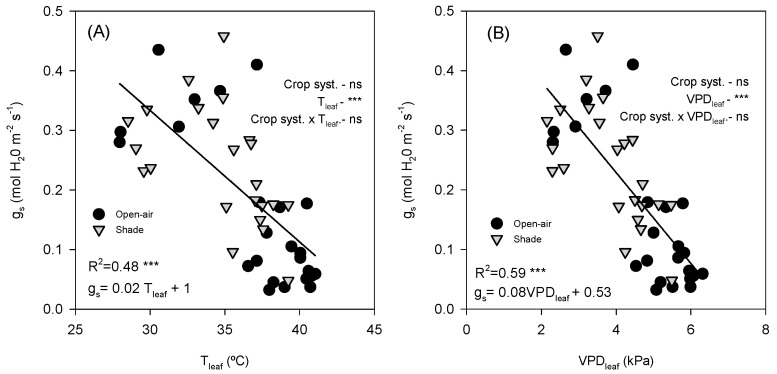
Relationship between leaf temperature (T_leaf_) and stomatal conductance (*g_s_*) (**A**) and between leaf vapour pressure deficit (VPD_leaf_) and *g_s_* (**B**). Data points are individual samples, linear regression lines are fitted, and *p*-values are reported. R^2^ indicates the square regression. Measurements were taken on a representative summer day (12 July 2022) in avocado trees grown under open-air (black dots) and shade (grey dots) conditions. The *p*-values for each main effect (crop system and *x*-variable) and their interaction are reported from two-way ANOVA, where *** and ‘ns’ indicate significant differences at *p* < 0.001 and no significant differences, respectively.

**Figure 7:**
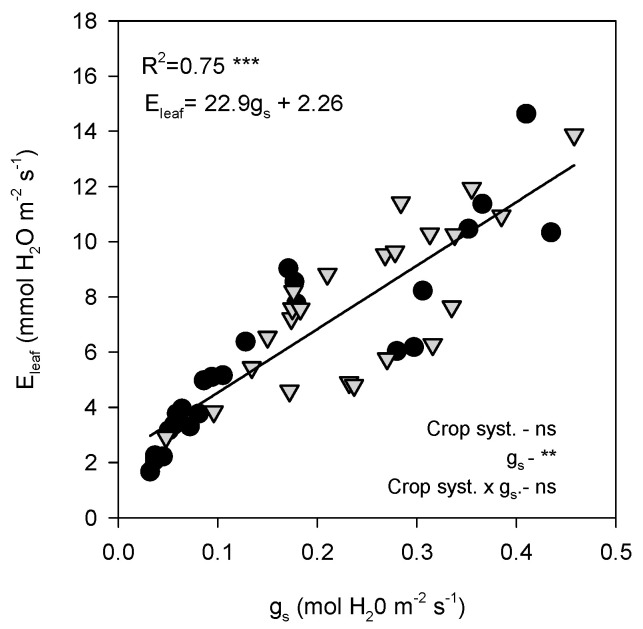
Relationship between stomatal conductance (*g_s_*) and leaf transpiration (*E_leaf_*). Data points are individual samples, the linear regression line is fitted, and *p*-values are reported. R^2^ indicates the square regression. Measurements were taken on a representative summer day (12 July 2022) in avocado trees grown under open-air (black dots) and shade (grey dots) conditions. The *p*-values for each main effect (crop system and *x*-variable) and their interaction are reported from *two-way* ANOVA, where **, *** and ‘ns’ indicate significant differences at *p* < 0.01, *p* < 0.001 and no significant differences, respectively.

Stomatal conductance showed a significant negative linear relationship with both T_leaf_ and VPD_leaf_. *g_s_* declined markedly as T_leaf_ increased (R^2^ = 0.47, *p* < 0.0001; Figure 6A). Similarly, *g_s_* decreased consistently with increasing VPD_leaf_ (R^2^ = 0.59, *p* < 0.0001; Figure 6B). Both open-air and shade-grown trees followed the same overall trend, as indicated by the covariance analysis, which showed no differences between treatments. Moreover, *E_leaf_* was strongly and positively correlated with *g_s_* (R^2^ = 0.75, *p* < 0.0001, Figure 7).

**Figure 8:**
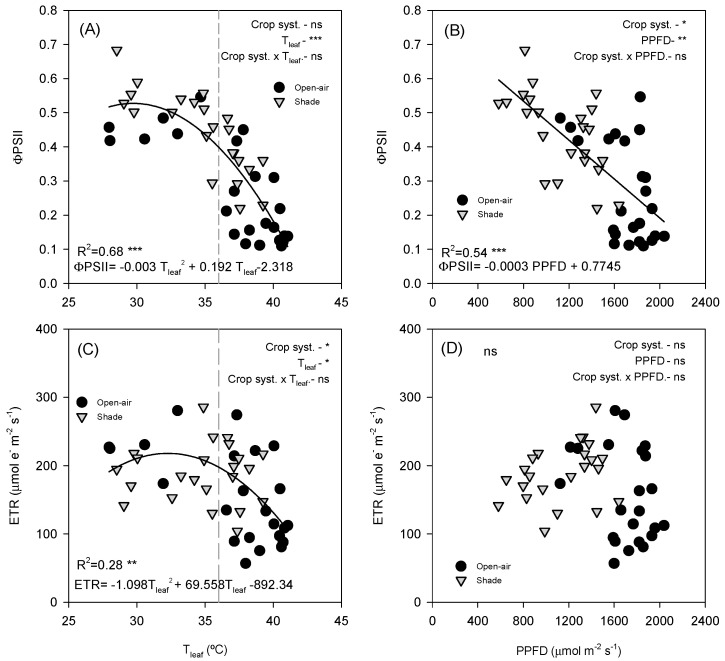
Relationship between leaf temperature (T_leaf_) and the effective quantum yield of PSII (ΦPSII, (**A**)) and electron transport rate (ETR, (**C**)), and between photosynthetic photon flux density (PPFD) and ΦPSII (**B**) and ETR (**D**). Data points are individual samples, multiple regression lines are fitted, and *p*-values are reported. R^2^ indicates the square regression. Measurements were taken on a representative summer day (12 July 2022) in avocado trees grown under open-air (black dots) and shade (grey dots) conditions. The *p*-values for each main effect (crop system and *x*-variable) and their interaction are reported from *two-way* ANOVA, where *, **, *** and ‘ns’ indicate significant differences at *p* < 0.05, *p* < 0.01, *p* < 0.001 and no significant differences, respectively. The vertical grey dashed line denotes the breakpoint at T_leaf_ = 36 °C.

ΦPSII was strongly affected by both leaf temperature and irradiance. A clear negative non-linear relationship (R^2^ = 0.68, *p* < 0.0001) was observed between ΦPSII and T_leaf_ (Figure 8A), with values decreasing sharply above ~36 °C. At T_leaf_ below 36 °C (grey dashed line), ΦPSII remained relatively stable, with values ranging between 0.4 and 0.6. However, above this threshold, a steep decline occurred, and ΦPSII dropped rapidly as the temperature increased, reaching minimum values of approximately 0.1–0.2 at the highest temperatures (>40 °C). According to the fitted breakpoint (36 °C), 70.8% of open-air data points were above this temperature, whereas only 43.5% of shaded observations surpassed it, highlighting the reduced exposure of shaded leaves to supra-optimal thermal conditions. Similarly, ΦPSII declined with increasing PPFD, showing a strong negative linear correlation (R^2^ = 0.54, *p* < 0.0001) (Figure 8B). At low PPFD (<800 µmol m^−2^ s^−1^, ΦPSII remained relatively high, but progressively declined as irradiance increased. Both open-air and shade-grown trees followed the same overall trend, as indicated by the covariance analysis, which showed no differences between treatments.

ETR exhibited a weaker but still significant dependence on T_leaf_ (Figure 8C; R^2^ = 0.25, *p* = 0.0018). The relationship followed a non-linear curve: ETR increased with temperature up to ~30–35 °C, reaching maximum values of ~300–400 µmol e^−^ m^−2^ s^−1^ before declining at higher temperatures. Both shade and open-air leaves exhibited similar maximum ETR values; in this case, the covariance analysis did not show any difference among treatments. In contrast, no significant relationship was detected between ETR and PPFD (Figure 8D). Across the PPFD data range, ETR values remained highly variable, with no consistent differences between shade and open-air conditions.

## 3. Discussion

Under Mediterranean climatic conditions, avocado trees are exposed to extreme environmental factors that severely constrain their physiological performance. High irradiance and temperature, together with elevated VPD, promote stomatal closure, reduce transpiration and photosynthetic efficiency, and increase the risk of photoinhibition and thermal damage. These stressors, acting synergistically during the summer, challenge the ability of avocado, a specie inherently adapted to mild, humid tropical environments, to maintain stable gas exchange and photochemical activity [6]. The results of this study demonstrate that shade netting effectively mitigates these limitations by moderating canopy temperature and incident radiation, leading to enhanced stomatal conductance and PSII efficiency. This microclimatic adjustment substantially reduced the intensity of environmental stress experienced by the trees, suggesting that shading is a practical and sustainable strategy to improve avocado adaptation to Mediterranean conditions.

The contrasting diurnal patterns of solar irradiance and VPD between summer and autumn highlight the critical role of microclimatic conditions in regulating avocado physiological responses. The daily patterns of environmental factors clearly separate summer and autumn (Figure 1). In summer, net solar radiation peaked above 900 W m^−2^ the temperature reached values around 35 °C, and VPD_air_ rose above 3.5 kPa (Figure 1A,C,D). By contrast, autumn conditions were considerably milder, with a maximum net solar radiation of 600 W m^−2^, T_air_ below 30 °C, and a VPD_air_ below 2 kPa. (Figure 1B,D,F) These seasonal differences were further amplified at the leaf level, where contrasting microclimatic conditions developed around the canopy in open-air and shaded trees (Figure 2). Under open-air conditions, the incident PPFD approached 2000 μmol m^−2^ s^−1^, and leaf temperatures exceeded 40 °C, while VPD_leaf_ reached values higher than 5 kPa (Figure 2), creating a combination of high light intensity and heat stress. By contrast, shading nets modified the leaf microclimate: shaded trees received a reduced PPFD, with maximum values around 1400 μmol m^−2^ s^−1^ (≈30% reduction; Figure 2A), lowered T_leaf_ by 3–5 °C, rarely surpassing 35 °C (Figure 2C), and decreased VPD_leaf_ by ~20–25% (from >5 to ~3.8–4.2 kPa; Figure 2E) Although the net-house did not consistently decrease air temperature or VPD_air_, the ≈30% attenuation of PPFD substantially reduced the radiative load on leaves, lowering T_leaf_ by 3–5 °C and consequently VPD_leaf_. This decoupling between leaf and air temperatures is expected from leaf energy-balance theory, which emphasises radiative forcing over convective exchange. Occasional morning/evening inversions in air temperature and episodes with higher VPD_air_ under shade are consistent with transient advection and limited ventilation typical of screenhouses. These microclimatic behaviours are well documented in screenhouse environments and arise from interactions between radiation screening and constrained air exchange [16].

In autumn, PPFD was still reduced by ~30% (∼1600→∼1100 μmol m^−2^ s^−1^), but T_leaf_ remained <30 °C in both treatments, and the decline in VPD_leaf_ was modest (Figure 2). Overall, these results confirm that the avocado, a specie adapted to shaded and humid environments, is particularly susceptible to the combined stress of high irradiance and temperature typical of Mediterranean summers, and that shading nets provide an effective management strategy to alleviate these constraints.

Despite the contrasting conditions between summer and autumn, Ψ_stem_ showed surprisingly little variation (Figure 3A,B), confirming that this parameter is not always a sensitive indicator of short-term water stress in avocado [6]. Avocado exhibits isohydric behaviour, maintaining relatively stable plant water status through strong stomatal regulation that limits transpiration when atmospheric demand increases [5]. In both open-air and shaded conditions, Ψ_stem_ declined progressively from morning to midday, following the typical diurnal pattern described for woody plants [12], but recovered slightly towards the evening. The similarity of Ψ_stem_ between treatments and seasons indicates that avocado trees maintained comparable overall water potentials yet achieved this through different stomatal strategies depending on environmental conditions. This isohydric behaviour was further supported by the analysis of ΔΨ_leaf_ and Δ*g_s_* (Figure 4). Throughout the summer day, ΔΨ_leaf_ remained close to zero, with only small fluctuations indicating that both crop systems maintained similar water potentials despite the contrasting microclimatic conditions. By contrast, Δ*g_s_* revealed a more dynamic response. While values were near zero in the morning, the difference between systems increased progressively during the afternoon, reaching positive values. This pattern indicates that shaded trees exhibited higher stomatal conductance when atmospheric demand and leaf temperature were at their peak (Figure 2). In autumn, the smaller environmental gradients were reflected in reduced diurnal variation in both ΔΨ_leaf_ and Δ*g_s_* (Figure 2), resulting in minor differences between systems (Figure 4).

Stomatal conductance and leaf transpiration were more responsive to environmental variations (Figure 3C–F). In summer, *g_s_* was higher in the early morning when VPD was still low (Figure 1E) but declined sharply as the day progressed and VPD_leaf_ exceeded 3.5 kPa, with T_leaf_ rising above 40 °C (Figure 2C and Figure 6). This decline in *g_s_*, consistent with strong stomatal control to reduce water loss under high evaporative demand, agrees with previous observations in other subtropical crops such as citrus [11]. The strong positive correlation between *g_s_* and *E_leaf_* (R^2^ = 0.75, < 0.0001; Figure 7) further confirmed the central role of stomata in regulating leaf water loss. However, while both treatments exhibited similar early morning *g_s_* values, open-air trees experienced a much steeper midday decline and failed to recover in the afternoon, in contrast to shaded trees, which maintained significantly higher *g_s_* and *E_leaf_* until late afternoon. This behaviour suggests that shading alleviated the severity of stomatal closure by maintaining cooler leaves (Figure 2C) and lower leaf-to-air vapor pressure gradients (Figure 2E). Such results align with those obtained in lime trees under shading nets, where higher *g_s_* and *E_leaf_* were also observed compared with open-air conditions [12]. In autumn, atmospheric demand remained below 2 kPa and T_leaf_ was below 30 °C (Figure 2D and Figure 3F), resulting in milder conditions for gas exchange. Both treatments exhibited typical midday peaks in *g_s_* and *E_leaf_*, similar to those previously reported in avocado under moderate climates [17]. Consequently, the benefits of shading were most evident in summer, when evaporative demand was high, while in autumn the effect was negligible. This seasonal pattern emphasises that shading primarily enhances avocado performance during periods of combined light and heat stress, improving stomatal regulation and maintaining active transpiration under otherwise restrictive conditions.

The sensitivity of avocado leaves to environmental stress was further evidenced by the behaviour of chlorophyll fluorescence parameters (Figure 5 and Figure 8). PSII photochemistry was strongly influenced by the combined effects of high irradiance and temperature, which act synergistically to reduce electron transport and the efficiency of light energy conversion. As irradiance increased beyond ~800 μmol m^−2^ s^−1^, ΦPSII declined markedly (Figure 8B), reflecting excess excitation energy that exceeded the capacity of carbon metabolism to use absorbed light, leading to photoinhibition [18]. The diurnal pattern of ΦPSII showed a typical decline at midday, when PPFD exceeded 1000 μmol m^−2^ s^−1^, followed by a recovery in the afternoon as irradiance decreased. This reversible midday depression indicates dynamic photoinhibition, a protective mechanism allowing the dissipation of excess light energy to prevent sustained damage to PSII [19,20].

Seasonal differences were nonetheless evident. In summer, the midday depression of ΦPSII was more pronounced than in autumn, likely due to the additional effect of elevated T_leaf_ (above 40 °C), which exacerbates the susceptibility of PSII to thermal deactivation [8,21]. The observed negative nonlinear relationship between ΦPSII and T_leaf_ (Figure 8A) supports this fact, showing a steep decline above 36 °C. Shading significantly moderated these effects by lowering both irradiance and canopy temperature, allowing shaded plants to maintain higher ΦPSII values throughout the day. Similar findings were reported in citrus [15], where shading effectively prevented photoinhibition by reducing excess light load, further confirming the protective role of shade nets under combined heat and light stress. In autumn, when environmental stress was reduced, overall fluorescence values were higher and differences between treatments were minimal (Figure 5B,D,F,H). This suggests that shading becomes less critical when temperature and radiation fall within the optimal range for avocado photosynthesis. Nevertheless, the marked improvements observed in summer confirm that shade nets provide an effective buffer against photochemical impairment under Mediterranean climatic conditions. The higher ΦPSII values under shading indicate improved coordination between energy absorption and utilization, reducing the excitation pressure on PSII and maintaining efficient photochemical performance.

The ETR exhibited a distinct thermal pattern compared with ΦPSII. In summer, ETR remained relatively stable throughout the day despite strong variations in irradiance and temperature, consistent with the observed temperature response curve, which showed an optimum between 35–38 °C (Figure 8C). This indicates that under field conditions, avocado leaves were already operating close to their thermal optimum. However, at higher temperatures, open-air leaves tended to exhibit a sharper decline in ETR, suggesting the onset of heat-induced inactivation of PSII or reduced re-oxidation of the plastoquinone pool [22,23]. In autumn, when temperatures were milder, ETR followed a clearer diurnal pattern, increasing with irradiance until midday and then declining thereafter. Notably, the lack of a consistent correlation between ETR and PPFD (Figure 8D), despite the marked decline of ΦPSII, implies that non-photochemical quenching mechanisms partially compensated for the excess energy under high light, maintaining the electron flow within safe limits.

Overall, these findings demonstrate that shading not only mitigated the risk of photoinhibition by reducing excess irradiance but also sustained more efficient electron transport and energy use in avocado leaves during summer. The enhanced photochemical stability observed under shaded conditions indicates that the combined reduction of leaf temperature and radiation load alleviated both thermal and photochemical stress, contributing to improved photosynthetic resilience in young avocado trees exposed to Mediterranean environments.

From a physiological perspective, shading improved both stomatal and photochemical performance of avocado trees under the combined stress of high irradiance and temperature typical of Mediterranean summers. Shaded trees maintained higher stomatal conductance and transpiration rates during the afternoon, while higher ΦPSII and ETR values reflected a more efficient photochemical function and a reduced risk of irreversible photodamage. This coordinated improvement between stomatal regulation and photochemistry indicates that shade nets enhance the overall photosynthetic resilience of avocado trees by optimizing the balance between CO_2_ diffusion and light energy use. In addition to the direct effects of temperature and radiation attenuation, differences in light quality and diffusion under the shade structure may have further contributed to the improved photochemical performance observed in shaded trees, as reported for other fruit crops cultivated under protective covers [24]. In contrast, during autumn, when environmental stress was lower and both irradiance and VPD remained moderate, shading provided only limited additional benefits, underscoring the seasonal dependence of its effectiveness.

Although shading has been previously studied in other fruit trees under Mediterranean conditions, such as citrus [12], apples [25], and grapevine [26], information for avocado remains scarce. The present work provides field-based evidence on how shade nets affect gas exchange and photochemical efficiency in avocado trees, a specie particularly sensitive to high temperatures and irradiance. Under conditions of high solar radiation (PPFD > 1500 µmol m^−2^ s^−1^ and elevated atmospheric demand (VPD_air_ > 3 kPa), shading reduced leaf temperature by approximately 3–5 °C, contributing to the maintenance of higher photochemical efficiency. In autumn, when these thresholds were seldom exceeded, shading showed no appreciable effect. These results collectively demonstrate that shading may represent a practical and sustainable agronomic strategy to mitigate the negative effects of summer heatwaves and radiation excess on avocado physiology in Mediterranean climates. By moderating canopy temperature and incident radiation, shade nets improve gas exchange parameters and enhance photosynthetic stability, while also increasing tree resilience against extreme climatic events. Considering current projections of increasing frequency and intensity of heat stress episodes under climate change scenarios [6,8], the adoption of shading systems may become a key adaptive practice to secure the long-term productivity and sustainability of avocado cultivation in Mediterranean regions. Nevertheless, as this study was conducted over a single growing season, further multi-year research is needed to confirm the consistency of these physiological patterns and to evaluate potential agronomic outcomes.

## 4. Materials and Methods

### 4.1. Experimental Site

This study was conducted in 2022 at a commercial orchard in Callosa d’En Sarrià, Alicante, Spain (38° 39′ N, 0° 07′ W, 247 m.a.s.l.). The region’s climate is Mediterranean, characterized by warm, dry summers and mild winters. The orchard consisted of 5-year-old avocado trees (cv. ‘Hass’) grafted onto ‘Duke 7’ rootstock, planted in a terraced crop system with a spacing of 6.0 m × 6.0 m between trees.

The irrigation system consisted of three drip lateral lines per tree row, spaced 0.5 m, with 36 pressure-compensated emitters with a flow rate of 1.6 L h^−1^ spaced 0.5 m. The trees were irrigated to fulfill plant water requirements throughout the experiment. Irrigation scheduling was based on the crop evapotranspiration (ET_c_) estimated by the single crop coefficient (K_c_) approach and reference crop evapotranspiration (ET_0_) [27]. The irrigation system included automatic control valves, an irrigation controller and flow metres to monitor irrigation volumes in each treatment. Trees were fertilized to cover the crop needs. Other agricultural practices were those often used by growers in the area.

### 4.2. Crop Conditions

**Figure 9:**
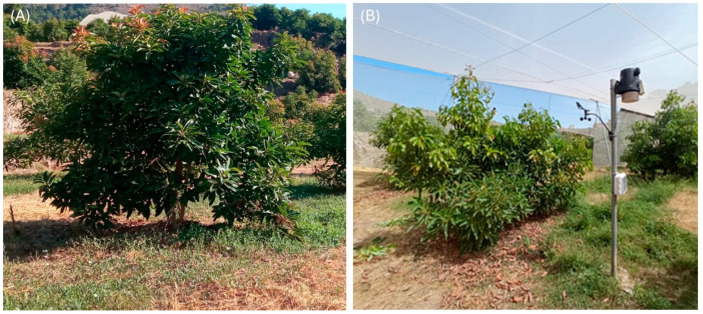
General view of avocado trees grown under open-air (**A**) and shade (**B**) conditions.

Two crop systems were established in 2021: open-air and shade (using a shade net) (Figure 9). Each treatment consisted of three blocks of 15 trees (45 trees per system). Only the two central trees of each block were selected for physiological measurements, while the remaining trees served as buffers to minimize border effects, and each tree was treated as an independent experimental unit. Both cases received the same amount of irrigation water and fertilizer. The shade net formed a net house 6 m high, covered with a white polyethylene monofilament of 1 mm thickness and a density of 6 × 6 strands cm^−2^ providing approximately 76% light transmittance. The structure was permanently installed throughout the experimental period.

### 4.3. Measurements

Two daily measurement cycles were conducted on representative summer (12 July 2022) and autumn (25 October 2022) days, representing high and low atmospheric water demand conditions, respectively. During each cycle, physiological and environmental parameters were recorded throughout the day to capture the diurnal dynamics under contrasting seasonal conditions.

#### 4.3.1. Environmental Conditions

Precipitation, air temperature (T_air_), and solar radiation were recorded in real-time by two automatic weather stations (Vantage Pro2, DAVIS Instrument Corporation, Hayward, Charlotte, NC, USA) located at both open-air and shade net systems. Data were recorded hourly. The air vapour pressure deficit (VPD_air_) for open-air and shade net was calculated hourly.

#### 4.3.2. Physiological Measurements

Plant measurements were taken at 1- to 2-h intervals from early morning to sunset. The stem water potential (Ψ_stem_) was measured with a pressure chamber (Soil Moisture Equipment Corp., Model 3000, Santa Barbara, CA, USA) in two mature leaves per tree. Fully expanded leaves from the shadow area were enclosed in an aluminium bag for at least 1 h before sampling.

Gas exchange parameters, including stomatal conductance (*g_s_*) and transpiration rate (*E_leaf_*), were measured on three sun-exposed fully expanded leaves per tree using an advanced portable porometer/fluorometer (LI-600, LI-COR, Lincoln, NE, USA). The chamber airflow rate was maintained at 500 μmol s^−1^. Chlorophyll fluorescence parameters, including maximum fluorescence in light-adapted leaves (*F_m_*′), steady-state fluorescence (*F_s_*), and the effective quantum yield of PSII [ΦPSII = (*F_m_*′ − *F_s_*)/*F_m_*′)] were determined. From these measurements, the electron flow rate (ETR, μmol e^−^ m^−2^ s^−1^) was calculated as ETR = ΦPSII × PPFD × leaf absorptivity coefficient × 0.5. Where PPFD (μmol m^−2^ s^−1^) is the photosynthetic photon flux density incident on the leaf; leaf absorptivity coefficient is the absorptance of the photosynthetic organ, and 0.5 is a correction factor for PPFD, assuming that half of the photons are absorbed by PSI and the other half by PSII. Leaf absorptance was set at 0.85, based on the assumption that approximately 85% of the photons incident on a standard leaf are delivered to the reaction centers. This is a typical value for C_3_ plants [28,29]. PPFD was recorded with the integrated quantum sensor of the LI-600 chamber, positioned at the leaf surface during each measurement. This measurement represents the instantaneous irradiance incident on the leaf at the time of gas exchange and fluorescence assessment. PPFD, leaf temperature (T_leaf_), and leaf vapour deficit pressure (VPD_leaf_) measurements were also taken with LI-600 simultaneously with the other measurements.

### 4.4. Statistical Analysis

Statistical analyses were performed using the Statsgraphics Centurion XVIII statistical package (Statistical Graphics Corp, Hendon, VA, USA). Data were analysed according to a *two-way* factorial repeated-measures ANOVA, with *System* (open-air vs. shade) as the between-subject factor and *Time of day* as the within-subject (repeated) factor, using individual trees as experimental units. Mauchly’s test of sphericity was applied, and when the assumption was violated (*p* > 0.05), the Greenhouse-Geisser correction was used to adjust the degrees of freedom. When a significant *System* × *Time* interaction was detected, differences between systems at each time were evaluated using the Duncan’s test (*p* < 0.05). The relationship between ΦPSII, ETR, and *g_s_* and leaf microclimate variables (PPFD and T_leaf_) were analysed using ANCOVA, with System (open-air vs shade) as a fixed factor and the environmental variable as a covariate. The equality of slopes (*System* × *covariate*) was tested and found to be non-significant, indicating that a common slope could be used for both systems. Consequently, linear or non-linear regression was fitted pooling both datasets.

## 5. Conclusions

This study demonstrates that shade netting significantly improves the physiological performance of young avocado trees grown under Mediterranean climatic conditions. Shading reduced incident radiation and canopy temperature, resulting in a more moderate microclimate that mitigated the combined effects of heat and light stress. As a result, shaded trees maintained higher stomatal conductance and transpiration during periods of maximum atmospheric demand, together with greater PSII efficiency and electron transport rates. These coordinated improvements indicate that shading enhances both stomatal and photochemical components of photosynthesis, thereby promoting better water relations and photoprotection during the summer.

From an agronomic perspective, the use of shade nets represents an effective and sustainable adaptation strategy to mitigate the detrimental effects of climate-induced heat and radiation extremes on avocado cultivation in Mediterranean regions. By reducing canopy stress and improving photosynthetic stability, shading can contribute to more resilient orchards. Future studies should focus on evaluating the long-term impact of shading on yield, fruit quality, and water-use efficiency, as well as optimizing shade intensity and colour for different cultivars and environmental contexts.

## Data Availability

All data are available from the corresponding author upon request.

## Abbreviations

The following abbreviations are used in this manuscript:

ANOVA	Analysis of variance
ET0	Reference evapotranspiration
ETc	Crop evapotranspiration
Eleaf	Leaf transpiration rate
ΦPSII	Quantum efficiency of photosystem II
Fm′	Maximum fluorescence in light-adapted leaves
Fs	Steady-state fluorescence
gs	Stomatal conductance
Kc	Crop coefficient
PPFD	Photosynthetic photon flux density
Tair	Air temperature
Tleaf	Leaf temperature
VPDair	Air vapour pressure deficit
VPDleaf	Leaf vapour pressure deficit
Ψstem	Stem water potential
